# Underreporting of implementation strategies and barriers in physical activity interventions for young people at risk of problematic substance use: a brief report

**DOI:** 10.1186/s43058-024-00578-9

**Published:** 2024-04-22

**Authors:** Lisa Klamert, Melinda Craike, Gillinder Bedi, Susan Kidd, Michaela C. Pascoe, Alexandra G. Parker

**Affiliations:** 1 Institute for Health and Sport, Victoria University, 70/104 Ballarat Rd, Footscray, VIC 3011 Australia; 2 Orygen, Parkville, VIC 3052 Australia; 3 Centre for Youth Mental Health, The University of Melbourne, Melbourne, VIC 3052 Australia; 4 Mitchell Institute for Education and Health Policy, Victoria University, Footscray, VIC 3011 Australia; 5 Acute Care Service, Tweed Byron Mental Health, Northern NSW Health District, Lismore, Australia

**Keywords:** Young people, Substance use, Physical activity, Adolescents, Early intervention, Implementation

## Abstract

**Background:**

Several studies have assessed whether physical activity interventions can reduce substance use in young people at risk of problematic substance use. This report identifies and describes the reporting of implementation characteristics within published studies of physical activity interventions for young people at risk of problematic substance use and provides recommendations for future reporting.

**Methods:**

Reported implementation strategies (including intervention manualization), barriers, implementation fidelity, and personnel acceptance were extracted from studies of physical activity interventions for young people aged 12–25 years at risk of problematic substance use that were included in a previous systematic review of intervention efficacy.

**Results:**

Implementation strategies were reported in less than half of the included studies (42.9%), implementation barriers in only 10.7% of studies, intervention fidelity in 21.4%, and personnel acceptance in a single study (3.6%).

**Conclusions:**

Results indicate insufficient reporting of implementation strategies, barriers, fidelity, and personnel acceptance. Consideration of implementation characteristics is essential for implementing physical activity interventions in practice. Inadequate or limited reporting of these characteristics may contribute to delayed uptake and adoption of evidence-based interventions in clinical practice. Recommendations to improve the reporting of implementation information include integrating standards for reporting implementation characteristics into existing reporting guidelines, developing an international taxonomy of implementation strategies, and upskilling intervention researchers in the fundamentals of implementation science.

**Supplementary Information:**

The online version contains supplementary material available at 10.1186/s43058-024-00578-9.

Contributions to the literature

- The lack of reporting of essential implementation characteristics in intervention studies may limit the uptake of potentially effective physical activity interventions for young people at risk of problematic substance use into practice.
- A strong collaboration or dual skilling is essential to upskilling researchers and bridging the gap between intervention and implementation research.
- The findings of this report highlight the importance of bringing together the fields of intervention and implementation research and adequate reporting of implementation characteristics to accelerate the availability of potentially effective interventions for young people with problematic substance use.

## Introduction

Adolescence and early adulthood, particularly the age group 12–25 [1], are peak periods for the emergence of psychiatric conditions and problematic substance use [2–4]. Problematic substance use and comorbid mental ill-health onsetting during this key developmental period pose critical risk factors for impaired life trajectories [2].

Physical activity (PA) and physical activity interventions represent one promising approach for early intervention for problematic substance use in young people [5, 6]. As the age range of 12–25 years is generally characterized by a decline in activity levels [7, 8], and more than 80% of young people currently do not reach recommended physical activity levels [9–11], this approach may also have benefits beyond substance use.

Early intervention and treatment are crucial to mitigate long-term consequences of substance use, mental ill-health, and sedentary behaviors in young people. Physical activity interventions have shown a beneficial effect on young people’s mental and physical health including substance use behavior [5, 12]; however, they are rarely implemented into practice [13].

To improve the uptake of physical activity interventions in clinical practice, a range of factors need to be considered and addressed. One way to support the uptake of physical activity interventions into practice is to ensure that essential implementation information—including implementation strategies and barriers that have been applied or identified within trialed interventions—is routinely reported in published studies. Shortcomings in reporting of essential implementation information reduce the likelihood that these interventions will be taken up in practice if proven effective (see also Rudd et al. [14]). Reporting strategies that were used to improve implementation, or barriers encountered in the respective study settings, could be used to inform further PA implementation studies, provide useful information for decision-makers, expedite the process of uptake and implementation of effective physical activity interventions into clinical practice, and thus reduce the time from research to public health impact [14, 15]. For this reason, integrating implementation thinking and implementation strategy into intervention studies should be a research priority within both PA intervention research, but also intervention research overall. Previous research indicates that less than 50% of effective interventions are being implemented into health services, and many face decades of delays from initial evidence to their implementation [16] which leads to delays in these interventions being available to individuals [17].

Although often considered the domain of implementation trials, the entire efficacy-effectiveness-implementation research spectrum may benefit from reporting of implementation factors and integration of discrete implementation strategies. Failure to consider implementation strategies from study initiation commonly leads to unplanned mid-course corrections [16]. Integrating implementation considerations early in the research process, as part of efficacy trials, may reduce these unplanned mid-course corrections, increase intervention fidelity, streamline progression to effectiveness research and subsequent implementation [18], and accelerate an intervention’s progression through the research spectrum.

To date, reviews of physical activity interventions for problematic substance use in young people have only considered the efficacy of interventions, rather than factors related to implementation. This report aimed to examine implementation strategies and barriers, implementation fidelity and acceptance of interventions among non-research personnel, and thus to highlight the importance of reporting implementation factors. The findings of this report will inform attempts to improve the reporting of intervention factors in future trials of physical activity interventions for young people at risk of problematic substance use and accelerate the uptake of evidence-based interventions into practice.

## Method

A systematic review of the effects of physical activity interventions was conducted between Nov 2020–Jan 2021 and updated in Nov 2022 [5]. Study eligibility was based on the intervention of interest (physical activity interventions including multimodal and acute studies applying cognitive, behavioral, and informational approaches), population of interest (young people aged 12–25 at risk of problematic substance use, defined as substance use that is associated with health and/or social problems and/or legal problems), outcomes of interest (substance use, physical activity, mental health), language (English), and study design (randomized-controlled trials (RCT) and non-RCT). The review included different formats and intervention approaches, including efficacy and effectiveness studies, and unimodal and multimodal approaches to provide a comprehensive review of existing evidence on physical activity interventions in this population. This report is a complimentary piece to Klamert et al. [5].

Due to the lack of international consensus regarding what comprises “critical” implementation characteristics, factors referring to implementability of healthcare interventions as reported by Klaic et al. [19] were extracted. These included implementation strategies (including sustainability and feasibility if reported), barriers (e.g., implementation context), implementation fidelity, and acceptance of the interventions among non-research personnel (for definitions see Table 1). Extracted implementation strategies were aligned with the Expert Recommendations for Implementing Change (ERIC) project, a compilation of internationally recognized implementation strategies [20]. Implementation barriers, implementation fidelity, and personnel acceptance were mapped onto the Consolidated Framework for Implementation Research (CFIR), a practical framework allowing the systematic assessment of implementation barriers and facilitators [21].

**Table 1:** Definitions of extracted implementation characteristics

Implementation characteristic	Definition
Implementation strategies	Arrangements of techniques that aim to enhance the adoption, implementation, and maintenance of clinical interventions and/or practice [15, 22] and facilitate change within the institutions or organizations in which interventions are implemented [23]
Implementation barriers	Factors obstructing the implementation of interventions [24]
Implementation fidelity	The degree to which an intervention is delivered as intended. High implementation fidelity is critical to the successful translation of evidence-based interventions into practice ([25], p. 1)
Personnel acceptance	Acceptance, intervention-knowledge, attitudes towards or adherence to the intervention among facilitating non-research personnel (i.e., providers) [26]; the extent to which an intervention is being perceived as agreeable, palatable by facilitators [27]

The report aimed to provide a brief overview of individual and service level factors associated with the implementability of healthcare interventions to highlight existing shortcomings, and the need for advancements in reporting standards relating to physical activity interventions for young people with substance use.

## Results

Twenty-eight studies were included in the review. Most of the interventions (92.9%) were delivered in educational or community settings. One or more implementation strategies were reported in 42.9% of the included studies (12/28), while 10.7% of the studies (3/28) reported one or more implementation barriers, 21.4% of studies reported on implementation fidelity (6/28), and 3.6% of studies (1/28) reported on acceptance of the intervention among involved non-research personnel.

Ninety-four percent of the extracted implementation strategies could be mapped onto 16 strategies included in the ERIC project. Fifty-seven implementation strategies included under the ERIC framework were not reported in any included study. The most frequently reported ERIC implementation strategies were *conduct ongoing training* (for peers, coaches, and staff) (25%, 7/28 studies) and *change service sites* (change service location to increase access) (14.3%, 4/28 studies). Five studies (17.9%) reported the development of manuals (i.e., *develop education materials* according to ERIC) based on the intervention or intervention elements. Two extracted strategies could not be assigned to ERIC implementation strategies (i.e., division of facilitation workload across multiple individuals to minimize facilitation burden).

Only four studies (14.3%) assessed implementation barriers and facilitators in line with proposed CFIR constructs, which are thought to be essential to the successful implementation of interventions. The most frequently assessed barriers were *location conditions* (Outer setting domain, assessed by 7.1%, 2/28 studies). Other barriers assessed included *local attitudes* (3.6%, 1/28 studies), *critical incidents* (3.6%, 1/28 studies), and *innovation deliverers* (3.6%, 1/28 studies). Implementation facilitators (personnel acceptance) were only reported in one included study. Forty-two essential implementation constructs according to the CFIR were not assessed in any study. For detailed implementation characteristics and their mapping onto ERIC and the CFIR, see Supplementary Table 1. For the pattern of reported implementation characteristics, see Table 2.

**Table 2:** Reporting of implementation characteristics of physical activity interventions for young people at risk for problematic substance use

Reference	Implementation strategies	Implementation barriers	Manualized	Implementation fidelity	Personnel acceptance
An et al. [28]	✓	X	X	X	X
Correia et al. [29]	X	X	X	X	X
Daniel et al. [30]	X	X	X	X	X
Daniel et al. [31]	X	X	X	X	X
Everson et al. [32]	X	X	X	X	X
Everson et al. [33]	X	X	X	X	X
Faulkner et al. [34]	X	X	X	X	X
Fishbein et al. [35]	✓	✓	✓	X	X
Ho et al. [36]	X	X	X	X	X
Blank et al. [37], Horn et al. [38], Horn et al. [39]	✓	X	✓	X	X
Janse Van Rensburg and Taylor [40]	X	X	X	X	X
Kerr et al. [41]	✓	X	✓	✓	X
Lane et al. [42]	X	X	✓	X	X
Melamed et al. [43]	✓	X	X	✓	X
Murphy et al. [44]	X	X	X	X	X
Oh and Taylor [45]	X	X	X	X	X
Parker et al. [46]	✓	X	✓	✓	X
Prapavessis et al. [47]	X	X	X	X	X
Prince et al. [48]	✓	X	X	X	X
Rotheram-Borus et al. [49]	✓	✓	X	✓	X
Scott and Myers [50]	X	X	X	X	X
Stanley et al. [51]	✓	✓	X	X	X
Taylor et al. [52], Taylor et al. [53]	X	X	X	X	X
Tesler et al. [54]	✓	X	X	X	X
Weinstock et al. [55]	X	X	X	X	X
Wilson et al. [56]	X	X	X	X	X
Weinstock et al. [57]	✓	X	X	✓	✓
Ybarra et al. [58]	✓	X	X	✓	X

## Discussion

This report outlines the reporting of implementation factors, including strategies, barriers, fidelity, and personnel acceptance, within studies of physical activity interventions for young people at heightened risk of problematic substance use. Extracted implementation factors were mapped onto existing implementation-focused systems and frameworks (ERIC, CFIR). Based on an efficacy-effectiveness review conducted by Klamert et al. [5], the reported implementation factors were extracted from 28 included studies. The review found that ERIC implementation strategies were under-reported as part of PA interventions; less than half of the identified studies reported implementation strategies that were used as part of the interventions. Implementation knowledge, which is essential to the successful implementation of an intervention according to the CFIR framework, such as implementation barriers, was only reported by just over a 10th of included studies. Implementation fidelity was reported by roughly one quarter. While the investigated studies included PA intervention studies only, findings of under-reporting may apply to other types of interventions more broadly, as indicated by an ongoing separation (rather than integration) of intervention development and implementation knowledge in healthcare research.

There was an overlap in extracted strategies with previous findings reported within the implementation of health interventions. This overlap included ongoing training courses in intervention delivery [59, 60], the use of train-the-trainer strategies, and accessing new funding [20, 59, 60]. Additional implementation strategies—not employed in studies covered in this review—have been identified in the literature more broadly [20].

Reported implementation barriers in this report aligned with those identified by Langley et al. [61] and Josyula and Lyle [62], namely, local conditions and attitudes (e.g., cultural environment) and increased workload on clinicians and administration as barriers (CFIR constructs: implementation team members, work infrastructure).

With reporting on personnel or provider acceptance limited to a single study, it was not possible to meaningfully compare findings with previous research evidence. Overall, personnel acceptance of and attitudes toward the implementation of evidence-based interventions have not been well studied within the international context [63].

Poor reporting of implementation strategies as part of research studies reduces the chances of evidence-based interventions being taken up into routine care and limits conclusions that can be drawn by decision-makers regarding the trialed interventions [16, 22].

One factor contributing to underreporting of implementation as part of intervention descriptions, but also impeding a priori integration of implementation considerations, is the inconsistent use of implementation terminology, even within the field of implementation science [64]. Consensus building and standardization of terms are essential to streamlining communication in these emerging fields [65, 66] and to the dissemination of implementation knowledge in research and practice [65]. Several attempts to develop international taxonomies of published implementation strategies [20, 67, 68], measure the effectiveness of individual strategies [69], and assess tailored implementation strategies for different contexts have been undertaken [20]. However, implementation strategies must not be just reported, but also “adequately reported,” i.e., reported in sufficient detail to allow for measurement and reproducibility of the strategy and/or its components in research or practice [22], to be useful and allow real-world application [70, 71].

Another factor contributing to poor reporting of implementation strategies may be the limited training of researchers studying new interventions in implementation science and the lack of direct consultation or collaboration of research teams investigating new health interventions with skilled implementation researchers (see also [72]). Proctor et al. [72] argue that this is due to the emerging nature of the field of implementation science, which continues to struggle with conceptual and methodological challenges.

### Limitations

There are several limitations to this report. Studies included were predominantly set in educational or community settings. For this reason, it is unclear whether the information extracted can be generalized to clinical settings.

Further, based on the shortcomings in reporting implementation characteristics in included studies, resulting in the extraction of only a small number of implementation characteristics, authors were not able to draw any conclusions regarding the effectiveness of reported implementation strategies and their impact on intervention success. Additionally, the authors’ decision to focus on a framework relating to the implementability may entail the exclusion of other implementation characteristics that are seen as relevant by other members of the scientific community.

### Recommendations

Based on current and previous evidence of underreporting of implementation characteristics in physical activity interventions for young people at risk of problematic substance use, we suggest the following recommendations for future research on PA interventions, but also healthcare interventions more broadly:

1. Upskill intervention researchers in the field of implementation. This could increase the likelihood of implementation considerations being included in the early stages of intervention development. A priori considerations in the early stages of research regarding the streamlining of evidence-based interventions from efficacy testing to implementation would likely lead to faster availability of effective interventions to clients.
2. Strengthen linkages between the fields of intervention research and implementation science through strong networks and multidisciplinary teams. While implementation science has developed from a need for effective interventions and treatments to be streamlined to clinical practice, both fields operate mostly independently with neither benefiting from discoveries in the respective other field in a timely manner.
3. Establish collaborations with and recruiting health care practitioners and relevant personnel (i.e., intervention facilitators) as research team members ([73], see also [74]).
4. Integrate existing taxonomies of implementation strategies subject to international consensus to decrease inconsistent terminology within the fields of implementation science.
5. Integrate reporting guidelines (including strategies, barriers, fidelity) (see also [22, 75]) into existing, internationally recognized reporting guidelines and checklists, such as the Template for intervention description and replication checklist (TIDieR) [13].
6. Establish implementation strategy as a research priority rather than a research addition or extension in the field of intervention development.

## Conclusion

There is limited reporting of implementation characteristics (including implementation strategies, barriers, intervention fidelity, and acceptance of interventions among non-research personnel) in studies of physical activity interventions for young people at heightened risk of problematic substance use. The underreporting may be related to several issues, including inconsistent implementation terminology, limited (a priori) integration of implementation considerations in intervention development, a limited number of researchers who are skilled in both implementation science and intervention development, and the absence of reporting standards for implementation characteristics. Exploration of these issues may reduce the underreporting of implementation characteristics in future publications.

Several recommendations to reduce underreporting and increase consideration of implementation characteristics as part of PA intervention research, but also healthcare intervention research overall have been made, including the development of internationally recognized standards for the reporting of implementation characteristics. Increased, high-quality reporting of this information is one factor that will likely contribute towards increasing the uptake of effective physical activity interventions in practice and streamlining intervention development from efficacy testing to implementation.

### Supplementary Information

**Additional file 1: Supplementary Table 1.** Detailed implementation characteristics of included studies.

## Data Availability

The data supporting the conclusions of this article are included within the article and its additional file or available upon request from the corresponding author.

## Abbreviation

PA: Physical activity
