# Supplementary material for: Underreporting of implementation strategies and barriers in physical activity interventions for young people at risk of problematic substance use: a brief report
Source: Implement Sci Commun. 2024 Apr 22;5:45. doi: 10.1186/s43058-024-00578-9 (PMC11036629; doi:10.1186/s43058-024-00578-9)
Supplement: Supplementary file 1 — Additional file 1: Supplementary Table 1. Detailed implementation characteristics of included studies. [file 43058_2024_578_MOESM1_ESM.docx]

**Supplementary Table 1**

*Detailed implementation characteristics of included studies*

| Reference | Implementation strategies | ERIC project (Powell et al., 2012) | Implementation barriers | Personnel acceptance | CFIR framework (Damschroder et al., 2022) | Implementation fidelity |
| --- | --- | --- | --- | --- | --- | --- |
| An et al. (2013) | - Weekly coach meetings - Partly monitored video messages/phone calls. - Peer training: Training in motivational   interviewing by counseling supervisor to ensure adherence of peers to protocols | - Conduct ongoing training - Develop and organize quality monitoring systems | Not specified | Not specified | n/A | Not specified |
| Correia et al. (2005) | Not specified | n/A | Not specified | Not specified | n/A | Not specified |
| Daniel et al. (2007) | Not specified | n/A | Not specified | Not specified | n/A | Not specified |
| Daniel et al. (2006) | Not specified | n/A | Not specified | Not specified | n/A | Not specified |
| Everson et al. (2006) | Not specified | n/A | Not specified | Not specified | n/A | Not specified |
| Everson et al. (2008) | Not specified | n/A | Not specified | Not specified | n/A | Not specified |
| Faulkner et al. (2010) | Not specified | n/A | Not specified | Not specified | n/A | Not specified |
| Fishbein et al. (2016) | - Implementation in small environment with personal support and community-based learning. - Teacher tasks balanced across teachers to minimize burden for teachers.   Consultation of expert clinical panel with implementation knowledge and experience regarding nontraditional schools. | - Change service sites - Use an implementation advisor | - Securing support for study from school staff   Minimizing study burden on school staff | Not specified | - Implementation team members (Domain: Individuals) - Work infrastructure (Domain: Inner setting) | Not specified |
| Ho et al. (2014) | Not specified | n/A | Not specified | Not specified | n/A | Not specified |
| Blank et al. (2017); Horn et al. (2013); Horn et al. (2011) | - Facilitator training: Facilitators identified by school staff/principals. - Condition-specific training provided by American Lung Association West Virginia and researchers. - N-O-T facilitator training: 1.5 days with focus on protocol. - BI training: 3 hours with focus on research protocol. | - Conduct ongoing training - Develop educational materials | Not specified | Not specified | n/A | Not specified |
| Kerr et al. (2013) | - Standardized curriculum training for facilitators. | - Develop educational materials - Conduct ongoing training | Not specified | Not specified | n/A | - Curriculum fidelity: Curriculum correctly implemented at 95% of sessions. - Fidelity assessment: 65% of curriculum sessions observed/ scored |
| Lane et al. (2012) | Not specified | n/A | Not specified | Not specified | n/A | Not specified |
| Melamed et al. (2022) | - NAVIGATE modules transferred into online format. - Training for health coaches in cognitive behavioral therapy (CBT) or motivational interviewing (MI). - Weekly supervision of coaches by registered psychotherapist. - Virtual care team to review client’s progression. - Use of a collaborative care model to overcome fragmentation barriers in mental health and primary care services. - Technology-enabled collaborative care (TECC) model to overcome barriers such as geographical barriers, time pressure and competing demands for the attention of providers. - Client communication limited to only one person (health coach) instead of entire healthcare team. | - Tailor strategies - Conduct ongoing training - Provide supervision - Change service sites - Create new clinical teams - Tailor strategies - Intervene with patients/consumers to enhance uptake and adherence | Not specified | Not specified | n/A | - E-platform collected program use metrics to measure participant e-platform use and health coaching services |
| Murphy et al. (1986) | Not specified | n/A | Not specified. | Not specified | n/A | Not specified |
| Oh and Taylor (2014) | Not specified | n/A | Not specified | Not specified | n/A | Not specified |
| Parker et al. (2016) | - Allocation to therapists according to their workload and availability. - Implementation directly into established youth mental health clinical services.   Intervention duration adapted to insurance funding for psychological therapies | - Develop resource sharing agreements - Place innovation on fee for service lists/formularies | Not specified | Not specified | n/A | - -Over 60% of participants received at least 3 sessions. - High fidelity of interventions as study was directly implemented in youth mental health clinical services and did not require access to specialized exercise equipment or off-site facilities. |
| Prapavessis et al. (2014) | Not specified | n/A | Not specified | Not specified | n/A | Not specified |
| Prince et al. (2020) | - Use of smartphones as convenient and functional tools: Two-way functionality of smartphones allows interactions between, researchers/ clinicians/ clients/ participants. | - Tailor strategies | Not specified | Not specified | n/A | Not specified |
| Rotheram-Borus et al. (2016) | - Training for soccer coaches: Training in HIV/substance abuse preventive interventions, trained in fundamental of behavior change. - Continuous monitoring and supervision from Stellenbosch University and Grassroot soccer (organization that implements soccer-based HIV prevention interventions across sub-Saharan Africa) to ensure correct implementation, support and fast and effective identification and elimination of any issues/concerns. - Monthly supervision meetings for coaches. - Community setting rather than medical setting. - Implementation of interventions within activities with sustainable funding stream (e.g., FIFA) - Soccer/vocational training as sites for HIV services. | - Create new clinical teams - Conduct ongoing trainings - Develop and organize quality monitoring systems - Provide supervision - Access new funding - Change service sites | - Challenging local, political situations and context - Gains in risk reduction challenged in the context of national unrest/job strikes | Not specified | - Local conditions (Domain: Outer setting) - Critical incidents (Domain: Outer setting) | High fidelity. |
| Scott and Myers (1988) | Not specified | n/A | Not specified | Not specified | n/A | Not specified |
| Stanley et al. (2017) | - Self-sustaining model: Peer to peer education model (University students can teach high school students at no cost). - Student teachers as agents of change in their own communities. | - Use train-the- trainer strategies - Identify and prepare champions | - Cultural setting of United Arab Emirates. | Not specified | - Local conditions (Outer setting domain) - Local attitudes (Domain: Outer setting) | Not specified |
| Janse Van Rensburg and Taylor (2008) | Not specified | n/A | Not specified | Not specified | n/A | Not specified |
| Taylor et al. (2006); Taylor et al. (2005) | Not specified | n/A | Not specified | Not specified | n/A | Not specified |
| Tesler et al. (2018) | - Implementation directly into established youth advancement center. - Shared resources of multiple expert organizations and municipality resulted in more comprehensive/widespread program. | - Change service sites - Develop resource sharing agreements | Not specified | Not specified | n/A | Not specified |
| Weinstock et al. (2014) | Not specified. | n/A | Not specified | Not specified | n/A | Not specified |
| Weinstock et al. (2016) | - Facilitator training: Workshop on MI and CM by outside expert, annual one-day refresher - Continuous supervision: Regular review of intervention binders/ audiotapes/ case discussion | - Provide ongoing training - Provide supervision | Not specified | Good | - Innovation deliverers (Domain: Implementation process) | - Fidelity assessed by 6 independent raters. - 127 randomly selected audiotapes assessed. - MI, EC, and CM items rated on a 7- point Likert scale. - Outcome: Interventions were rated as having good therapist adherence/ competence. |
| Wilson et al. (2018) | Not specified | n/A | Not specified | Not specified | n/A | Not specified |
| (Ybarra et al., 2013) | - Technological feasibility of program tested prior to study start. Findings of feasibility testing were fed back into the program. - Involvement of youth advisory groups in intervention development | - Conduct small tests of change - Use advisory boards and workgroups | Not specified | Not specified | n/A | Good |
